# P02-017 - Periodic fever syndrome masquerading as eczema…

**DOI:** 10.1186/1546-0096-11-S1-A124

**Published:** 2013-11-08

**Authors:** TV Nguyen, J Sugarman, K Leslie

**Affiliations:** 1 Dermatology, University of California, San Francisco, San Francisco, USA

## Introduction

“Periodic Fever Syndrome Masquerading as Eczematous Dermatitis: Report of a D12E Mutation in *TNFRSF1A*”

## Case Report

A 22 year-old woman presented with a six-year history of periodic fevers, skin and joint disease. She presented with an intermittent, intensely pruritic red rash on the torso and extremities, associated with recurrent non-axial joint pain and swelling, and periodic fevers lasting for days to weeks. These symptoms were triggered by exposure to heat, cold weather, dry air, and sweat. She denied eye or gastrointestinal involvement. Physical exam revealed generalized, poorly demarcated, excoriated, erythematous macules and patches. Skin histology showed minimal psoriasiform hyperplasia and mixed perivascular infiltrates. Serum C-reactive protein, erythrocyte sedimentation rate, and immunoglobulin D levels were within normal range. Antinuclear antibody and rheumatoid factor tests were negative. Genetic testing revealed a heterozygous nucleotide substitution in the *TNFRSF1A* gene causing Tumor Necrosis Factor-Associated Periodic Syndrome (TRAPS). This mutation, namely D12E, results in substitution of an Aspartic acid with a Glutamic acid. Her mother is an asymptomatic heterozygote. After failing high-potency topical steroids and antihistamines, she was started on etanercept 100 mg weekly. Initial improvement of symptoms was dramatic, but the effects waned after four months of therapy. She was then switched to adalimumab 40 mg every other week and reported marked reduction of her skin manifestation and fevers. However, her condition relapsed after six months of using adalimumab. At that point, adalimumab was replaced with anakinra, an interleukin 1 (IL-1) receptor antagonist, and the patient has been reporting significant improvement in the past four months.

## Discussion

Patients with *TNFRSF1A* mutations affecting the cysteine residues are predisposed to a more severe phenotype, with fevers lasting for 23 days on average and many inflammatory symptoms.[1] Thus far only three cases of D12E carriers have been described: one with short-lived fevers and almost no other manifestations,[1] one with TRAPS phenotype,[2] and one also harboring a homozygous mutation for and presenting with the Familial Mediterranean Fever syndrome phenotype.[3] Our patient’s disease is likely related to aberrant innate immunity. The fact that she and her mother carry the same mutation, yet only one is symptomatic, suggests incomplete penetrance of the allele. Response to inhibition of IL-1 signaling is reassuring.

## Disclosure of interest

None declared.
